# Anti-Hyperalgesic Properties of Menthol and Pulegone

**DOI:** 10.3389/fphar.2021.753873

**Published:** 2021-11-30

**Authors:** Louis Hilfiger, Zélie Triaux, Christophe Marcic, Eléa Héberlé, Fathi Emhemmed, Pascal Darbon, Eric Marchioni, Hugues Petitjean, Alexandre Charlet

**Affiliations:** ^1^ Benephyt, Strasbourg, France; ^2^ Centre National de la Recherche Scientifique, University of Strasbourg, Institute of Cellular and Integrative Neurosciences, INCI UPR3212, Strasbourg, France; ^3^ Centre National de la Recherche Scientifique, University of Strasbourg, Institut Pluridisciplinaire Hubert Curien, IPHC UMR, Strasbourg, France

**Keywords:** pulegone, pain, rodent, menthol, inflammatory pain, monoterpene

## Abstract

**Context:** Menthol, the main monoterpene found in *Mentha piperita* L. (*M. piperita*) is known to modulate nociceptive threshold and is present in different curative preparations that reduce sensory hypersensitivities in pain conditions. While for pulegone, a menthol-like monoterpene, only a limited number of studies focus on its putative analgesic effects, pulegone is the most abundant monoterpene present in *Calamintha nepeta* (L.) Savi (*C. nepeta*), a plant of the Lamiaceae family used in traditional medicine to alleviate rheumatic disorders, which counts amongst chronic inflammatory diseases.

**Objectives:** Here, we analyzed the monoterpenes composition of *C. nepeta* and *M. piperita*. We then compared the putative anti-hyperalgesic effects of the main monoterpenes found, menthol and pulegone, in acute inflammatory pain conditions.

**Methods:** *C. nepeta* and *M. piperita* extracts were obtained through pressurized liquid extraction and analyzed by gas chromatography-mass spectrometry. The *in vitro* anti-inflammatory activity of menthol or pulegone was evaluated by measuring the secretion of the tumour necrosis factor alpha (TNF α) from LPS-stimulated THP-1 cells. The *in vivo* anti-hyperalgesic effects of menthol and pulegone were tested on a rat inflammatory pain model.

**Results:** Pulegone and menthol are the most abundant monoterpene found in *C. nepeta* (49.41%) and *M. piperita* (42.85%) extracts, respectively. *In vitro*, both pulegone and menthol act as strong anti-inflammatory molecules, with EC50 values of 1.2 ± 0.2 and 1.5 ± 0.1 mM, respectively, and exert cytotoxicity with EC50 values of 6.6 ± 0.3 and 3.5 ± 0.2 mM, respectively. *In vivo*, 100 mg/kg pulegone exerts a transient anti-hyperalgesic effect on both mechanical (pulegone: 274.25 ± 68.89 g, *n* = 8; vehicle: 160.88 ± 35.17 g, *n* = 8, *p* < 0.0001), thermal heat (pulegone: 4.09 ± 0.62 s, *n* = 8; vehicle: 2.25 ± 0.34 s, *n* = 8, *p* < 0.0001), and cold (pulegone: 2.25 ± 1.28 score, *n* = 8; vehicle: 4.75 ± 1.04 score, *n* = 8, *p* = 0.0003). In a similar way, 100 mg/kg menthol exerts a transient anti-hyperalgesic effect on both mechanical (mechanical: menthol: 281.63 ± 45.52 g, *n* = 8; vehicle: 166.25 ± 35.4 g, *n* = 8, *p* < 0.0001) and thermal heat (menthol: 3.65 ± 0.88 s, *n* = 8; vehicle: 2.19 ± 0.26 s, *n* = 8, <0.0001).

**Conclusion:** Here, we show that both pulegone and menthol are anti-inflammatory and anti-hyperalgesic monoterpenes. These results might open the path towards new compound mixes to alleviate the pain sensation.

## Introduction

Medicinal plants amongst the Lamiaceae family, including *Mentha piperita* L. (*M. piperita*) and *Calamintha nepeta* (L.) Savi (*C. nepeta*), have been used in traditional medicine since the ancient Greeks (Carović-Stanko et al., 2016) and during the Renaissance to alleviate rheumatoid disorders and chronic pain in the upper valley of the Rhine in Europe (Adams et al., 2009). *C. nepeta* was first described by Carl Linneus as “Melissa nepeta.” Since 1753, it has been replaced in the *Calamintha*, *Thymus*, *Satureja*, *or Clinopodium* genera. This plant grows as a perennial herb native to Europe and the Mediterranean region. It bears ovate, opposite gray-green leaves, white to pink tubular, two-lipped flowers and is very fragrant when crushed according to the World Checklist of Selected Plant Families (Govaerts, 2020). Adams and collaborators found mentions of usage of *C. nepeta*, a synonym for *Clinopodium nepeta* (L.) Kuntze, as a treatment of pain and rheumatism in two major herbals of the 17th century (Adams et al., 2009). Mattioli’s herbal advised the drinking of boiled calamint against gout and tough slime (Mattioli, 1554). Moreover, Fuchs’ herbal advises the use of an external application of leaves against hip pain (Fuchs, 1543). Since then, it has not been used much by modern herbalists, making it an interesting plant to re-investigate.

The main bioactive compounds in Lamiaceae plants are secondary metabolites including terpenoids, alkaloids, and phenolic compounds (Tissier et al., 2015). With about 70,000 structures described (Harborne, 1995), terpenoids are considered the largest family of natural compounds (Vavitsas et al., 2018). Those natural compounds are classified by the number of isoprene units containe in their chemical structure. Monoterpenes, composed of two isoprene units, are the main components of essential oils and are responsible for their diverse well-known biological activities (Salakhutdinov et al., 2017). In general, monoterpenes are derived from geranyl diphosphate (GPP) which is a product of the head-to-tail coupling of the primary metabolites isopentenyl diphosphate (IPP) and dimethylallyl diphosphate (DMAPP) (Rehman et al., 2016). Less than 50 monoterpenes have been described as potential analgesic (Guimarães et al., 2013) and/or anti-inflammatory molecules (De Cássia Da Silveira E Sá et al., 2013). However, menthol, the major monoterpene found in *M. piperita* (Ristorcelli et al., 1996), is known to modulate nociceptive threshold and is present in different curative preparations that reduce sensory hypersensitivities in pain type such as visceral pain (Galeotti et al., 2002), inflammatory pain (Pan et al., 2012) as well as neuropathic pain (Patel et al., 2014). One of the known mechanisms enabling menthol to act as a pain-killer is the inhibition of neural activity via the desensibilization of the TRPM8 channels expressed by the nociceptive sensory fiber endings (Pan et al., 2012; Liu et al., 2013).

Pulegone, a major monoterpene found in the essential oil of *C. nepeta* (Božović et al., 2017; Li et al., 2017), is obtained from GPP through limonene and piperitone, isopulegone being its direct precursor (Turner and Croteau, 2004). Pulegone is a precursor in the biosynthesis of menthol through menthone (Li et al., 2017). This monoterpene acts as an avian repellents through the modulation of the TRPM8 and TRPA1 channels expressed by bird sensory neurons (Majikina et al., 2018). In spite of the similarities between menthol and pulegone, the analgesic potential of pulegone was only studied in one visceral pain model (de Sousa et al., 2007) and one spontaneous pain model (de Sousa et al., 2011). Despite these observations, there is not much information on neither the putative effect of a curative pulegone treatment nor on nociceptive modalities on which it may act.

Interestingly, a recent review (Quintans et al., 2019) lists several terpenes that modulate the activity of cytokines, including the tumour necrosis factor (TNF-α), a cytokine involved in the primary onset of inflammatory responses maintenance and in its chronicity (Basbaum et al., 2009). Menthol and pulegone are cited within this list. Despite this potent anti-inflammatory action, only recent works explore the potential analgesic actions of terpenes (Guimarães et al., 2013; Yang et al., 2019a, 2019b; Perri et al., 2020; Bai et al., 2021; Wojtunik-Kulesza et al., 2021). Therefore, in the present study, we hypothesized that pulegone might have potent analgesic action on peripheral painful inflammatory sensitization, and tested its *in vitro* anti-inflammatory effect and cytotoxicity and its *in vivo* anti-hyperalgesic effects in the Freund’s Complete Adjuvant (CFA)-induced inflammatory pain model.

## Results

### *Mentha piperita* L. and *Calamintha nepeta* L. Extracts Analysis

**FIGURE 1:**
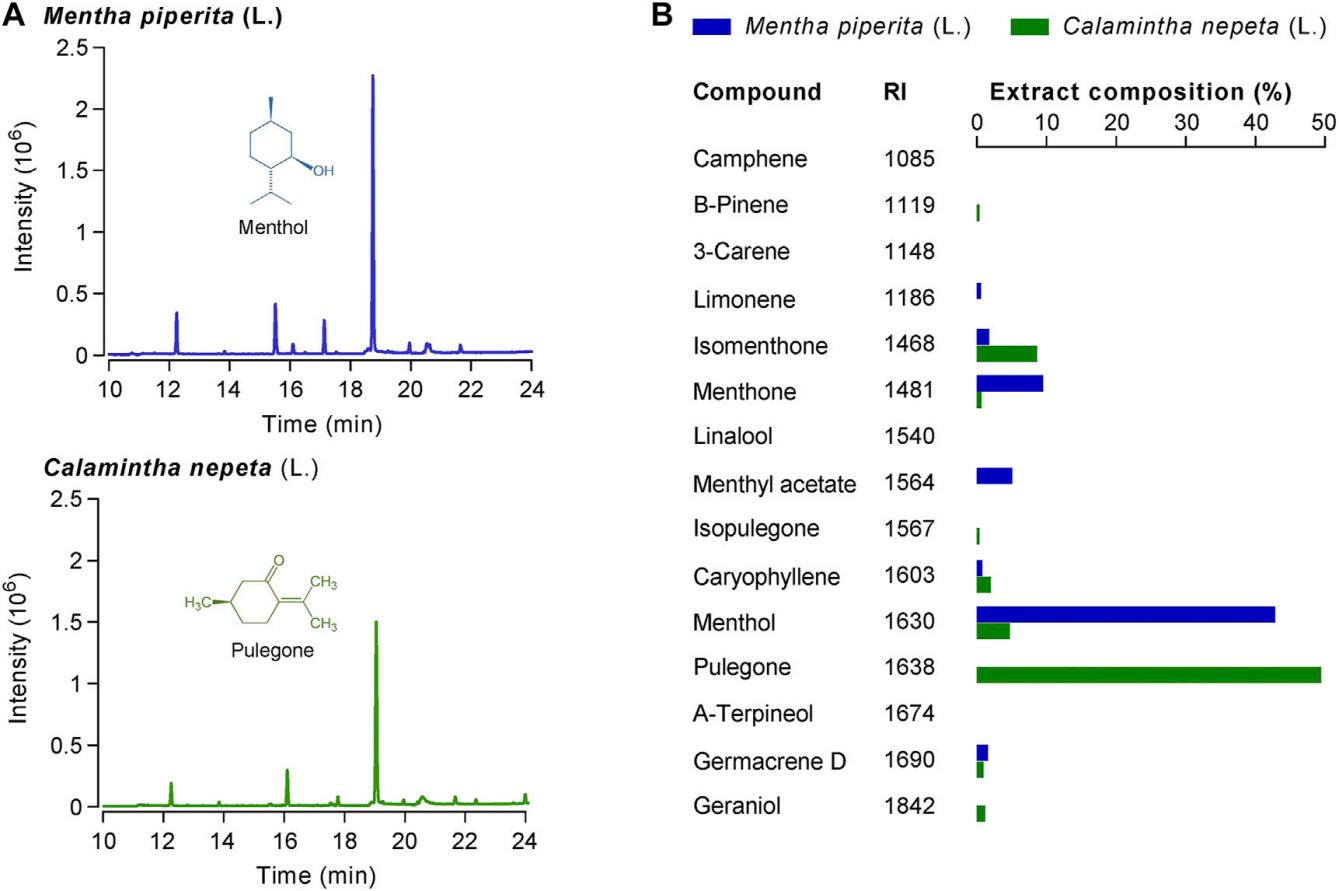
**(A)** Chromatograms of *Calamintha nepeta* (L.) Savi and *Mentha piperita* L. PLE extracts 10-fold diluted were analyzed by GC-MS. On both chromatograms, the main components of each extract are identified: pulegone (T_R_ = 19.04 min) for *C. nepeta* and menthol (T_R_ = 18.75 min) for *M. piperita*. **(B)** Extract composition (in %) together with retention index (RI) of each found compound.

*M. piperita* and *C. nepeta* extracts were obtained by pressurized liquid extraction (PLE) and analyzed by gas chromatography-mass spectrometry (GC-MS) (Figure 1A; Supplementary Figure S1). Overall, 11 volatile compounds were identified in the *M. piperita* extract (Figure 1B) and 10 in the *C. nepeta* PLE extract. As expected, the main constituent of the *C. nepeta* extract was pulegone (49.41%), while the one of the *M. piperita* extract was menthol (42.85%). The presence of isopulegone in the *C. nepeta* extract (Figure 1B), indicates that the isomerase conversion was not complete. In the same extract, menthol, isomenthone, and menthone can be found at lower concentrations than pulegone. Given the absence of pulegone detection in *M. piperita* extracts, its analgesic effects in traditional medicine cannot be attributed to pulegone. On the other hand, it raises the question of pulegone putative analgesic action, as it is the principal monoterpene found in *C. nepeta* (Adams et al., 2009).

Considering these results, we then restricted our investigation to the potential anti-inflammatory and analgesic properties of these two major compounds, pulegone and menthol.

### Menthol and Pulegone Display *In Vitro* Anti-inflammatory Effects

**FIGURE 2:**
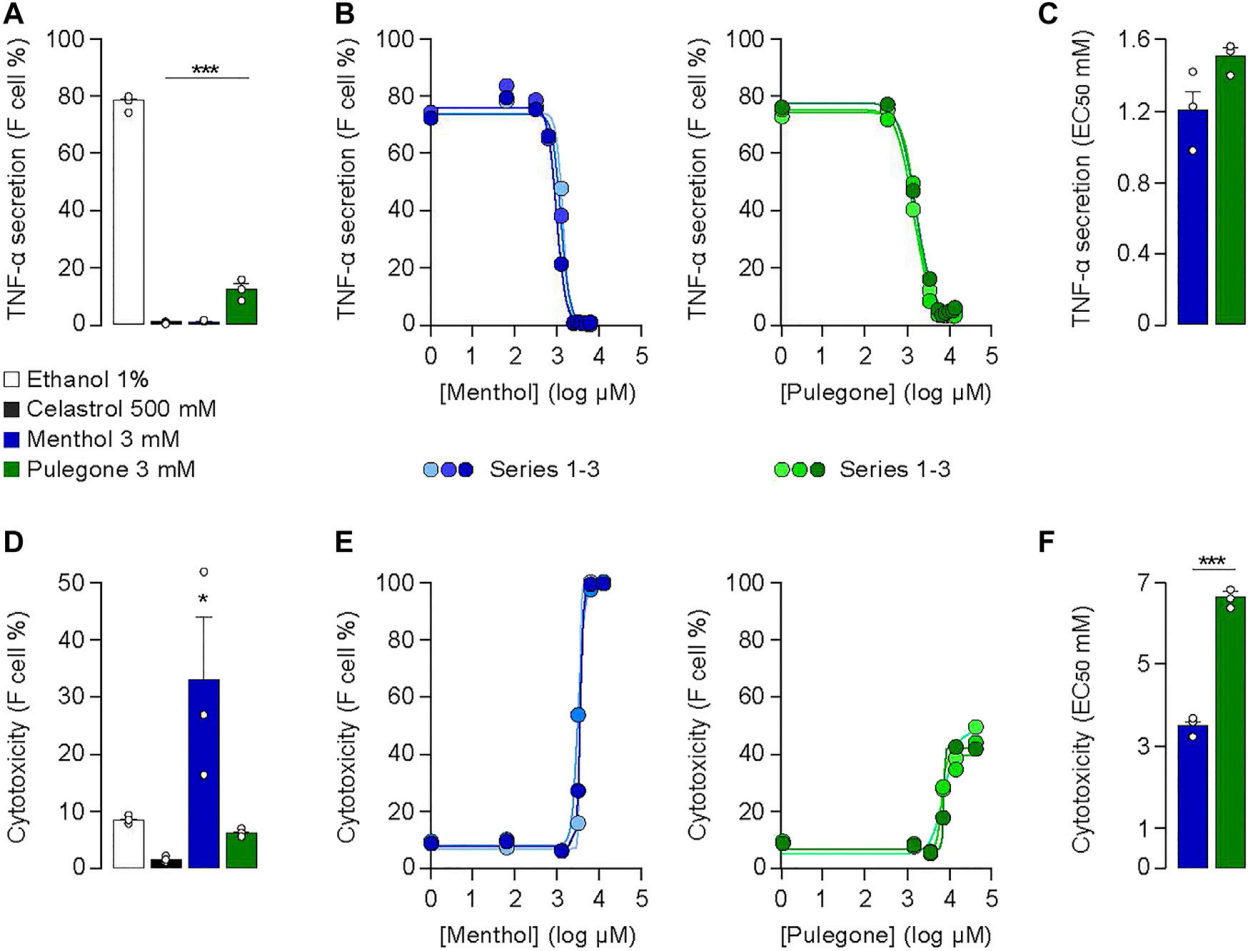
**(A)** Anti-inflammatory activities and **(D)** cytotoxicities of pulegone and menthol at 3 mM in 1% ethanol after 4 h LPS-stimulation of THP-1 cells. Experiments (*n* = 3) were conducted on the same 96-well plate to compare to the same negative control (1% ethanol) (***p < 0.001; **p < 0.01; *p < 0.05). Celastrol at 500 mM was used as the positive control. **(B)** One-way ANOVA F(3;8) = 1,031.6, *p* < 0.001, **(E)** one-way ANOVA F(3;8) = 6.22 *p* = 0.05). Dose-response curves of **(B)** anti-inflammatory activity and **(E)** cytotoxicity of pulegone and menthol after 4 h LPS-stimulation of THP-1 cells. Concentration range: 0.03–13 mM. Each concentration was tested in triplicates on the same 96-well plate. **(C,F)** EC_50_ values calculated by the dose-response curves of **(C)** the anti-inflammatory activity and **(F)** cytotoxicity of menthol and pulegone. Ratios of the EC_50_ value of the anti-inflammatory activity over the EC_50_ value of the cytotoxicity for menthol (in blue) and pulegone (in green) compared with a *t*-test (***p < 0.001; **p < 0.01; *p < 0.05).

The *in vitro* anti-inflammatory activity of menthol or pulegone was evaluated by measuring the secretion of the TNF-α from THP-1 cells after 4 h incubation with either menthol or pulegone (Figures 2A–C; Supplementary Figure S2). We used LPS to induce production of TNF-α, an effect that is blocked by celastrol (500 mM), a triterpene used as a positive control of the inhibition of the secretion of TNF-α (Allison et al., 2001); Figure 2A; ethanol 1%: 78.3 ± 0.7% vs. celastrol 500 mM: 1.1 ± 0.1%, *n* = 3, F(3;8)= 1,031.6, Dunnett Test *p* < 0.001). Interestingly, we observed a similar reduction of the secretion of TNF-α after incubation with menthol 3 mM (reduced to 1.2 ± 0.05% *n* = 3, *p* < 0.001) or pulegone 3 mM (reduced to 12.5 ± 2.2% *n* = 3, *p* < 0.001) (Figure 2A). We then evaluated the *in vitro* cytotoxicity of those different compounds. Interestingly, pulegone 3 mM presents a low cytotoxicity, comparable to the one of celastrol 500 mM, our positive control (5.6 ± 0.2% *n* = 3, and 1.4 ± 0.2% *n* = 3, respectively; Figure 2D). However, menthol 3 mM displayed a significant cytotoxicity (32.5 ± 11.1%, *n* = 3, F(3;8) = 6.22, Dunnett Test *p* = 0.017) compared to vehicle (1% ethanol; 8.4 ± 0.3%, *n* = 3, *p* = 0.038; Figure 2D). Therefore, pulegone showed a promising anti-inflammatory activity conjugated with low cytotoxicity.

To refine this gross characterization, we performed dose-response curves (0.03–13 mM) of the anti-inflammatory activity (Figure 2B) and the cytotoxicity (Figure 2E) of menthol and pulegone. The anti-inflammatory activity curve of each compound presents, notably, a similar distribution and an EC_50_ value in the same range (menthol: 1.5 ± 0.1 mM, n = 3; pulegone: 1.2 ± 0.2 mM, *n*= 3; Figure 2C; *t*-Test DF= 4, *p* = 0.09), suggesting equivalent anti-inflammatory activities of both, menthol and pulegone. However, the cytotoxicity curves were significantly different, showing a 100% cells death rate with a concentration of 12.8 mM of menthol (Figure 2E), while a similar pulegone concentration induced only 45.1% cells death rate. In accordance with this observation, the EC_50_ of the cytotoxicity was 3.5 ± 0.2 mM for menthol and 6.6 ± 0.3 mM for pulegone (*t*-Test DF= 4, *p* = 0.0001; Figure 2F). Taken together, those results indicate that THP-1 cells, which are among the first cells involved in the inflammation process, can withstand a higher concentration of pulegone than menthol.

To further characterize the effect on menthol and pulegone, we then investigated their potential pain-killer effects *in vivo* on nociceptive thresholds.

### Menthol and Pulegone Induce *In Vivo* Anti-hyperalgesia

**FIGURE 3:**
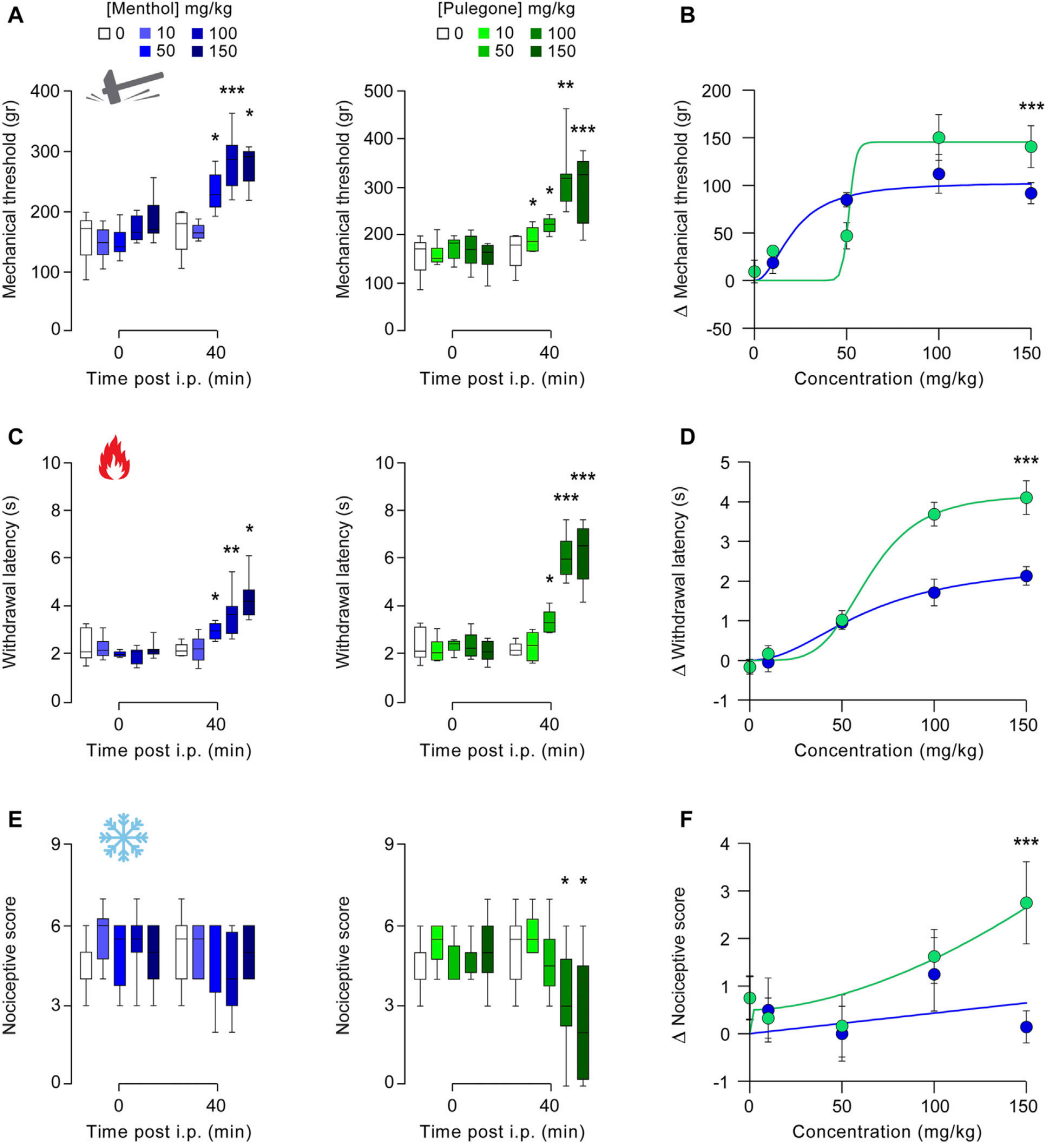
Dose-response of the analgesic properties of menthol and pulegone on CFA-induced inflammatory pain model. Effect of menthol 10 (*n* = 6), 50 (*n* = 6), 100 (*n* = 8), and 150 mg/kg (*n* = 7) and pulegone 10 (*n* = 6), 50 (*n* = 6), 100 (*n* = 8), and 150 mg/kg (*n* = 8) or their vehicle (*n* = 8) measured 40 min after i.p. injection on mechanical **(A)**, thermal heat **(C)**, and thermal cold. **(E)** CFA-induced hyperalgesia. Dose-response distribution fits for menthol and pulegone on mechanical **(B)**, thermal heat **(D)**, and thermal cold **(F)**. Data are expressed as mean ± SD. Asterisks indicate statistical significance (***p < 0.001; **p < 0.01; *p < 0.05) using Kruskal-Wallis test followed by Dunn’s multiple comparisons *post-hoc* test to compare each delta with the delta of the vehicle, to compare each treatment depending on the time post i.p. (0 vs. 40 min) it is a paired *t*-test or a Wilcoxon test, depending on the data’s normal distribution.

We first performed a dose-response curve of the putative analgesic effects of menthol and pulegone, measured on mechanical, thermal heat, and thermal cold sensitivities, 40 min after a single intraperitoneal (i.p.) injection itself performed 24 h after intra-plantar injection of complete Freund adjuvant (Hilfiger et al., 2020) (CFA; Figure 3;). CFA-model, similarly to other inflammatory-induced pain models, induces mechanical, thermal heat, and cold hyperalgesia, with a plateau in hyperalgesia observed between 24 and 48 h following CFA injection (Hilfiger et al., 2020). We selected several doses of monoterpene treatments within a range previously published in protocols (de Sousa et al., 2011; Pan et al., 2012).

First, both menthol and pulegone induced dose-dependent increase of the mechanical threshold (Control vs. menthol: DF= 4, *p* = 0.0002; vs. pulegone: DF= 4, *p* < 0.001, Kruskal-Wallis and Dunn’s comparisons Figures 3A,B), up to a plateau value obtained after 50 mg/kg for menthol (84.83 ± 17.87 ∆g, *n* = 6; *p* = 0.0162 DF= 4, *p* < 0.001, Kruskal-Wallis and Dunn’s comparisons) and 100 mg/kg for pulegone (150.2 ± 67.79 ∆g, *n* = 8; *p* = 0.0007). Interestingly, while anti-hyperalgesic effects of menthol start at lower concentration than the one of pulegone (50 mg/kg vs. 100 mg/kg), the plateau effect induced by pulegone was slightly higher than the one induced by menthol. Secondly, both menthol and pulegone induced a dose-dependent increase of thermal heat withdrawal latency (Control vs. menthol: DF= 4, *p* < 0.0001; vs. pulegone: DF= 4, *p* =0.003, Kruskal-Wallis and Dunn’s comparisons, Figures 3C,D), up to a plateau value obtained after 100 mg/kg (menthol: 1.713 ± 0.951 ∆s, *n* = 8; *p* = 0.0016; pulegone 3.688 ± 0.852 ∆s, *n* = 8; *p* = 0.0005). As previously, the anti-hyperalgesic effect of pulegone was significantly higher than the one induced by menthol (*p* = 0.0019). Thirdly, pulegone but not menthol induced a significant decrease in thermal cold acetone score (Control vs. menthol: DF= 4, *p* < 0.0001; vs. pulegone: DF= 4; *p* =0.1789, Kruskal-Wallis and Dunn’s comparisons, Figures 3E,F) following 100 mg/kg injection (menthol: −1.25 ± 2.19 ∆score, *n* = 8; *p* = 0.0546; pulegone −1.63 ± 1.6 ∆score, *n* = 8; *p* = 0.0256). Finally, the anti-hyperalgesic effects of both terpenes were compared through their respective dose-response distribution, and showed that pulegone induced a significantly higher anti-hyperalgesic effect than menthol on all tested modalities (*p* < 0.001; Figures 3B,D,F). Interestingly, none of those observations were reproduced on contra-lateral paw (i.e., paw without inflammatory sensitization), suggesting an absence of potential deleterious anti-nociceptive effect (Supplementary Figure S3).

**FIGURE 4:**
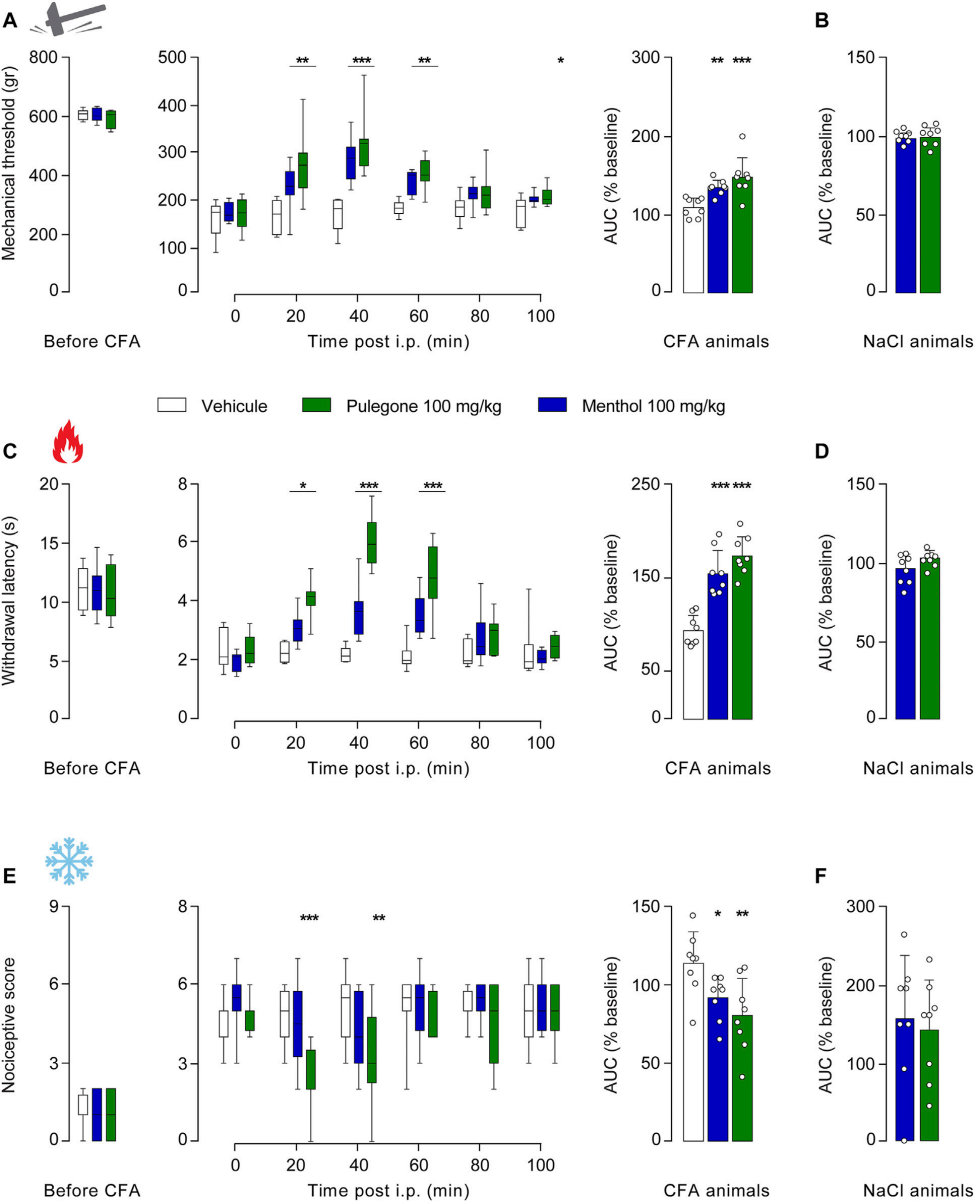
Time-course of the analgesic properties of menthol and pulegone on CFA-induced inflammatory pain model. Baseline, time-course, and relative-to-baseline AUC (%) of the effects of i.p. menthol 100 mg/kg (*n* = 8), pulegone 100 mg/kg (*n* = 8), or the vehicle (*n* = 8) on CFA-induced mechanical **(A)**, thermal heat **(C),** and thermal cold **(E)** hyperalgesia. Relative-to-baseline AUC (%) of the effects of i.p. menthol 100 mg/kg (*n* = 8) and pulegone 100 mg/kg (*n* = 8) on mechanical **(B)**, thermal heat **(D),** and thermal cold **(F)** sensitivities of NaCl-injected hindpaw. Data are expressed as mean ± SD. Asterisks indicate statistical significance (***p < 0.001; **p < 0.01; *p < 0.05) using two-way ANOVA followed by Dunnett or Sidak multiple comparisons test for the time course, for the AUC is a one-way ANOVA followed by a Holm-Sidak multiple comparisons test or *t*-test, depending on the data’s normal distribution giving the following statistic: mechanical: two-way ANOVA F(5;110) = 45.47, *p* < 0.0001 and one-way ANOVA F(2;28) = 11.61, *p* < 0.0001; thermal heat: two-way ANOVA F(5;110) = 53.74, *p* < 0.0001 and one-way ANOVA F(2;28) = 24.47, *p* < 0.0001 and thermal cold two-way ANOVA F(5;110) = 11.75, *p* < 0.0001 and one-way ANOVA F(2;28) = 4.3, *p* = 0.0128.

Along with this dose-response that allowed us to determine the optimal dose of i.p. terpene injection with regard to their anti-hyperalgesia effect (100 mg/kg), we next aimed to evaluate its duration. We thus monitored the mechanical, thermal heat, and thermal cold sensitivities every 20 min following i.p. injection of either menthol or pulegone at 100 mg/kg, carboxymethyl cellulose (CMC, 1%) in NaCl (0.9%) as a control vehicle (Figure 4; Supplementary Figure S4). The comparison of the AUC will be used to assess the global effect of each treatment.

Although, pulegone induced an anti-hyperalgesic effect on all measured modalities as soon as 20 min after i.p. injection on mechanical threshold (pulegone: 274.25 ± 68.89 g, *n* = 8; vehicle: 160.88 ± 35.17 g, *n* = 8, F (5, 110) = 45.47, *p* < 0.0001, two-way RM ANOVA and Dunnett’s multiple comparisons, Figures 4A,B), as well on thermal heat threshold (pulegone: 4.09 ± 0.62 s, *n* = 8; vehicle: 2.25 ± 0.34 s, *n* = 8, F (5, 110) = 53.74, *p* < 0.0001, two-way RM ANOVA with Dunnett’s multiple comparisons test, Figures 4C,D), and also on thermal cold threshold (pulegone: 2.25 ± 1.28 score, *n* = 8; vehicle: 4.75 ± 1.04 score, *n* = 8, F (5, 110) = 11.75, *p* < 0.0001, RM ANOVA with Dunnett’s multiple comparisons test, Figures 4E,F), significant effects of menthol were observable only after 40 min post i.p. injection (mechanical: menthol: 281.63 ± 45.52 g, *n* = 8; vehicle: 166.25 ± 35.4 g, *n* = 8, 8, F (5, 110) = 45.47, *p* < 0.0001 two-way RM ANOVA and Dunnett’s multiple comparisons Figure 4A; thermal heat: menthol: 3.65 ± 0.88 s, *n* = 8; vehicle: 2.19 ± 0.26 s, *n* = 8, F (5, 110) = 53.74, *p* < 0.000, two-way RM ANOVA with Dunnett’s multiple comparisons test Figure 4C).

Nevertheless, the effects of both terpenes fade 80 min after i.p. injection (mechanical: pulegone: 212 ± 42.46 g, *n* = 8; menthol: 209.38 ± 25.08 g, *n* = 8; versus vehicle: 179.92 ± 26.55 g, *n* = 8, *p* = 0.1979 and *p* = 0.2579, respectively, Figure 4A; thermal heat: pulegone: 2.84 ± 0.64 s, *n* = 8; menthol: 2.76 ± 0.89 s, *n* = 8; vehicle: 2.19 ± 0.44 s, *n* = 8, *p* = 0.1155 (pulegone), *p* = 0.1951 (menthol), Figure 4C; and thermal cold: pulegone: 4.5 ± 1.6 score, *n* = 8; vehicle: 5.13 ± 0.64 score, *n* = 8, *p* = 0.6344, Figure 4E).

Of note, menthol only slightly alleviated thermal cold hyperalgesia after i.p. injection (thermal cold: menthol: 91.39 ± 13.93%, *n* = 8, *p* = 0.2696, Figure 4E). Importantly, those effects were totally absent in non-hyperalgesic animals that received NaCl intraplantar injection (Figures 4B,D,F) as well as in the CFA contra-lateral hindpaw (Supplementary Figure S4), illustrating an anti-hyperalgesic action of those terpenes in absence of anti-nociceptive action. In addition, it is interesting to note that the anti-hyperalgesic effects of pulegone seem to be higher than those of menthol, especially for thermal heat modality (Figure 4D).

Altogether, these results indicate that a single i.p. injection of either menthol or pulegone exerts a significant and medium-lasting anti-hyperalgesic action on mechanical, thermal heat, and thermal cold sensitivities in an inflammatory-induced pain hypersensitivity rat model.

### Menthol and Pulegone Are Devoid of *In Vivo* Locomotor Side Effects

Given that monoterpenes might have strong unwilling side-effects when injected at high doses (pulegone >200 mg/kg) (de Sousa et al., 2011; Silveira et al., 2014), we performed several controls to assess that the previous experiments are free of motor reflex bias. To do so, we quantified hindpaw diameter as well as the motor activity of the rats after the i.p. injection of either pulegone or menthol, at the highest dose of 150 mg/kg (Figure 3), using two different experimental paradigms.

**FIGURE 5:**
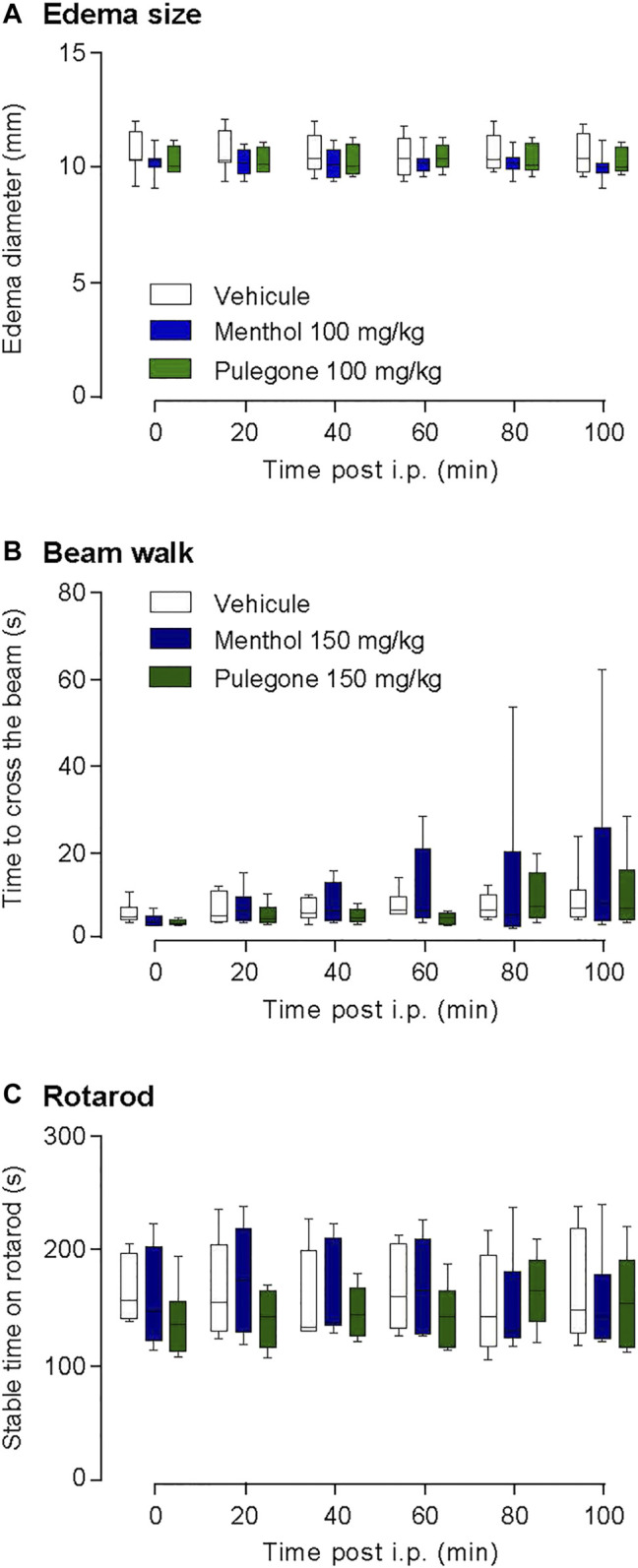
Control of terpenes effect on edema and locomotor activity. **(A)** Time-course of the effects of i.p. menthol 100 mg/kg (*n* = 8), pulegone 100 mg/kg (*n* = 8), or the vehicle (*n* = 8) on CFA-induced edema size (two-way ANOVA F(5;75) = 1.643, *p* =0.1526). Time-course of the locomotor activity after an i.p. injection (Vehicle, *n* = 6; Menthol (150 mg/kg), *n* = 6; Pulegone (150 mg/kg), *n* = 6) tested with the **(B)** rotarod two-way ANOVA F(5;75) = 0.2535, *p* =0.9367 and the **(C)** beam walk two-way ANOVA F(5;75) = 3.34, *p* =0.089. Data are expressed as mean ± SD. No statistical significance was observed, using two-way ANOVA followed by Dunnett multiple comparisons *post-hoc* test.

In a few of monoterpenes known to have anti-inflammatory action (De Cássia Da Silveira E Sá et al., 2013; Souza et al., 2014), the hindpaw edema diameter due to CFA was not reduced by neither pulegone nor menthol i.p. injection (vehicle, 10.55 ± 0.86 mm, *n* = 8; menthol, 10.18 ± 0.66 mm, *n* = 8; pulegone, 10.29 ± 0.66 mm, *n* = 8; Figure 5A). This suggests that an acute terpene injection, at this stage of the CFA-induced inflammation, may have no or limited anti-inflammatory effect but rather acts directly on nociceptive thresholds through a mechanism that remains to be explained.

In order to verify that neither menthol nor pulegone i.p. injection affected the locomotor ability of the rats and consequently alters the assessment of the nociceptive thresholds during the experimentation, we monitored their locomotor functions after i.p. injection using the beam walk assay (Figure 5B) as well as the rotarod assay (Figure 5C). In those two tests, both menthol and pulegone failed to impair the locomotor abilities of the animals.

## Discussion

In the present study, we confirmed that the monoterpenes menthol and pulegone are the major monoterpenes present in *M. piperita* and *C. nepeta* extracts, respectively (Figure 1). We found that menthol and pulegone initiate very similar *in vitro* anti-inflammatory activities, while pulegone seems to display only limited *in vitro* cytotoxic effects on TPH-1 macrophages (Figure 2). Finally, we revealed anti-hyperalgesic properties of both menthol and pulegone, with a significant advantage for pulegone (Figures 3, 4), without negative locomotor side effects (Figure 5).

Our results are in agreement with previous investigations, confirming that menthol and pulegone are the main monoterpenes present in *M. piperita* and *C. nepeta* extracts, respectively (Ristorcelli et al., 1996; Božović et al., 2017; Li et al., 2017). In addition, we confirmed the anti-inflammatory properties of pulegone and menthol, showing that both menthol and pulegone blocked the release of TNF-α from the LPS-stimulated THP-1 cell line (Figure 2B). Indeed, this result is in line with a previous report demonstrating on another macrophage cell line that menthol can suppress the LPS-induced TNF-a release (Shahid et al., 2018). A similar effect was observed with pulegone (Choi et al., 2018; Yang et al., 2019b, Yang et al., 2019a). Further experiments are required to take advantage of the significantly weaker cytotoxicity of pulegone observed in the THP-1 cell line (Figure 2B) especially to reduce pro-inflammatory cytokines release by these cells under chronic pain conditions.

Investigating the analgesic properties of menthol and pulegone *in vitro* an *in vivo* on CFA-induced inflammatory hypersalgesia, we found, as expected, that both menthol (Pan et al., 2012; Liu et al., 2013) and pulegone were able to alleviate the mechanical and thermal heat hyperalgesia. However, only pulegone was able to alleviate thermal cold hyperalgesia. In addition, we showed that, at similar doses, pulegone has a higher anti-hyperalgesic potency than menthol, in both intensity and duration (Figures 3, 4).

While the underlying mechanisms remain unknown, we propose a framework based on several hypotheses that remain to be explored. After peripheral inflammation, central sensitization leads to molecular and cellular changes in the central nervous systems where affinity and expression of channels and receptors like the gamma-aminobutyric acid receptor type A (GABA-A) increase and directly contribute to pain hypersensitivity (Latremoliere and Woolf, 2009). Thanks to their small molecular size and lipophilic properties, monoterpenes can cross the blood-brain-barrier (BBB) (Zhang et al., 2015; Weston-Green, 2019). Interestingly, other reports have shown that both menthol and pulegone can potentialize GABA-A receptors currents responsible for the inhibition of neuronal activity (Corvalán et al., 2009; Tong and Coats, 2012; Lau et al., 2014) and in this case, contribute to analgesia. Another possible pathway for menthol and pulegone to act as pain-killers might be to directly modulate the electrical activity of nociceptors, through the desensitization of their receptors targets involved in the sustenance of hypersensitivity states. Indeed, TRPM8 and TRPA1 are particularly involved in the detection and transduction of cold temperatures and noxious stimulations into neuronal activity (Basbaum et al., 2009; McKemy, 2013). TRPA1, a known target of menthol and pulegone (Majikina et al., 2018), is a cation channel contributing to transduce noxious temperatures above 42°C and involved in the propagation of nociceptive mechanical stimulation after inflammation (Brierley et al., 2011). In addition, it has been demonstrated that TRPA1 upregulation contributes to peripheral sensitization during inflammation and is responsible for both mechanical pain and thermal hypersensitivity (Petrus et al., 2007). Furthermore, high concentrations of agonists, such as monoterpenes, at the vicinity of sensory neurons can induce desensitization of both TRPM8 (Sarria et al., 2011; Liu and Rohacs, 2020) and TRPA1 (Akopian et al., 2007; Kühn et al., 2009). Therefore, one can hypothesize that monoterpenes could induce desensitization of TRPM8 and TRPA1, leading to a decrease of mechanical and cold thresholds, eventually leading to analgesia. Comparable mechanisms exists for the TRP vanilloid 1 (TRPV1), a channel involved in the detection of nociceptive hot temperatures and activated by the red chili pepper active compound, capsaicin (Basbaum et al., 2009): Plantar capsaicin injection in naïve animals induces nocifensive reactions and pain hypersensitivities (Petitjean et al., 2014, 2020), while long exposure to capsaicin induces long-term desensitization of the TRPV1 contributing to capsaicin-induced analgesia.

If these concepts are valid and transposable to humans, monoterpenes might be key candidates for clinical use due to their capability to cross the BBB and interact with many different molecular targets during inflammation, with no or limited locomotor side effects, these compounds might be key candidates for clinical use. This work may lead to the development of easy to take drugs, to be administered every few hours in order to limit pain symptoms.

In conclusion, we showed that pulegone has a significantly higher effect than menthol on nociceptive sensory thresholds in an inflammatory-induced pain hypersensitivity rat model. We establish for the first time that pulegone acts as an anti-hyperalgesic compound in the setting of inflammatory pain conditions on mechanical and thermal pain hypersensitivities. Such properties of pulegone suggest that traditional treatments based on *C. nepeta* preparations have indeed the potential to reduce chronic inflammation and reduce pain conditions as it was described in ancient herbals (Fuchs, 1543; Mattioli, 1554). This work thus supports the interest and potential of ethnopharmacological research for the discovery of new innovative treatments to alleviate chronic pain conditions.

## Materials and Methods

All the protocols, tests, and use of living animals were performed in accordance with European committee council Direction, from the regional ethic committee (Comité Régional d’Ethique en Matière d’Expérimentation Animale de Strasbourg, CREMEAS) and with authorization from French Department of Agriculture (APAFIS# 19006-2019020714109922 v3).

### Reagents and Chemicals

Ethanol was purchased from Sigma-Aldrich (Steinheim, Germany). Milli-Q water (18.2 MΩ) was generated by Millipore synergy system (Molsheim, France). Nitrogen was of 4.5 grade and helium of 6.0 grade (Sol France, Saint-Ouen l’Aumone, France). Pulegone (97%) and menthol (99%) were analytical standards obtained from Sigma-Aldrich (Steinheim, Germany). RPMI 1640 cell culture media was obtained from ATCC (LGC Standards, Molsheim, France). Penicilin and streptomycin were purchased from Cambrex Bio Science (St Beauzire, France). Fetal bovine serum (FBS) was obtained from Lonza BioWhittaker (Fisher Scientific, Illkirch, France). Lipopolysaccharide (LPS) from *Salmonella abortus equi* and celastrol were purchased from Sigma-Aldrich (Steinheim, Germany). Propidium iodide was obtained from Miltenyi Biotec Inc. (Auburn, AL, United States). For *in vivo* biological assays, terpenes were emulsified in warmed (37°C) carboxymethyl cellulose (CMC, 1%) - NaCl (0.9%) and administered at the temperature of 37°C. Pulegone, menthol, or vehicle was injected intraperitoneally (i.p.).

### Plant Material

*Plants. Calamintha nepeta* (L.) Savi and *Mentha piperita* L. were obtained from the Ledermann-Mutschler nursery (Krautergersheim, France). Upon reception, leaves and stems of the plants were chopped and dried at room temperature and out of the light until constant weight was obtained, indicating that the drying process was done. Dry plants were then cryogenically grounded (6,870 Freeze/Mill, Spex CertiPrep, Stanmore, Royaume-Uni) into fine powders which were stored at 4°C and protected from the light before extraction.

### Pressurized Liquid Extraction

Plant extracts were obtained by solid/liquid extraction under pressure using an ASE-350 system (Dionex, Sunnyvale, CA, United States). For the extraction, 3 g of finely grounded plant powder mixed with chemically inert Fontainebleau sand (previously heated at 600°C for 4 h and stored at room temperature) was placed into a 10 ml stainless steel extraction cell. Two 27 mm cellulose filters (Dionex, Sunnyvale, CA, United States) were placed one at the bottom and one at the top of the extraction cell. The extraction cell was then subjected to one cycle of extraction at 125°C under 100 bars for 7 min in the static extraction mode. The extraction solvent was a 50/50% (v/v) mixture of water and ethanol. The volume of the collected extract was about 15 ml. Between runs, the system was washed with 20 ml of the extraction solvent.

### Gas Chromatography-Mass Spectrometer (GC-MS) Conditions

Plant extracts were analyzed on a 450-GC/240-MS system (Varian, Les Ulis, France) equipped with a DB-WAX capillary column (60 m × 0.25 mm × 0.15 µm) (Agilent Technologies, Les Ulis, France). Two microliters of the extract were injected in a split/splitless injector at 210°C and carried through the column by helium carrier gas (99.9999%) at 1 ml/min. The following temperature program was applied to the column oven to allow the separation of the different compounds: hold for 1 min at 40°C, increased to 100°C at 10°C/min, increased to 130°C at 5°C/min, increased to 150°C at 10°C/min, increased to 180°C at 5°C/min, heated to 230°C at 10°C/min, and finally held isothermal at 230°C for 5 min. For the MS parameters, the transfer line temperature was set to 200°C and the ion source at 150°C. The mass spectrometer was operated in electron impact (EI) mode, and the ionizing electron energy was set to 70 eV. The mass spectra were registered in a full scan acquisition mode in the range of 50–200 m*/z*. Peaks were identified by referring the mass spectra to the NIST (National Institute of Standards and Technology) mass spectral database and by comparing their retention time to the one of the analytical standard.

### Drug Preparation

For *in vitro* assays, purified pulegone and menthol (Sigma-Aldrich (Steinheim, Germany) were prepared with a final 1% ethanol concentration for cell incubation.

For *in vivo* assays, purified pulegone and menthol (Sigma-Aldrich (Steinheim, Germany) were emulsified in warmed (37°C) carboxymethyl cellulose solution (CMC, 1%; in NaCl 0.9%) for subsequent intraperitoneal (i.p.) injections.

### Cell Culture

THP-1 cell line, acute monocytic leukemia cells, was purchased from the American Type Culture Collection (ATCC TIB-202, LGC Standard, Molsheim, France). The cells were maintained in RPMI-1640 (ATCC) medium supplemented with 10% (v/v) fetal bovine serum and 1% (v/v) mixture of penicillin (1,000 UI/ml, Gibco^TM^) and streptomycin (1,000 μg/ml, Gibco ^TM^). Cells were grown in 75 cm^2^ flasks in a humidified atmosphere with 5% CO_2_ at 37°C and were replicated every 2–3 days before reaching a concentration of 1 × 10^6^ cells/ml. Cells were sub-cultured at a concentration of approximately 2–3 × 10^5^ cells/ml by adding fresh media to the flask.

### TNF-α Secretion Assay

The TNF-α secreted by the THP-1 cells was measured in the culture medium using an assay kit (TNF-α secretion assay, Miltenyi Biotech, United States). TNF-α secretion was obtained by LPS activation of the THP-1 cells. THP-1 (180 µl) in suspension in the media at a concentration of 3 × 10^5^ cells/ml was seeded in a 96-well plate. LPS (20 µl) was added in each well to reach a final concentration of 1 μg/ml. In appropriate wells, 20 µl of plant extract dissolved in 90/10 water/ethanol were added to the cell media. For the evaluation of the activities of the standards, 2 µl of a standard solution in ethanol were added to reach specified final concentrations (from 0.03 to 13 mM). Positive and negative controls were included in the TNF-α secretion assay. Celastrol, a triterpenoid known for its TNF-α inhibitor capacities in *in vitro* tests (Allison et al., 2001), was used as a positive control at a final concentration of 0.5 µM. LPS-stimulated cells with 1% of ethanol were used as a negative control. The 96-well plate was then incubated at 37°C in 5% CO_2_ incubator for 2 h. After incubation, 2 µl of each antibody of the assay kit (catch reagent and detection reagent) were added in each well. The 96-well plate was once more incubated at 37°C in 5% CO_2_ incubator for 2 h. Propidium iodide (2 µl) was then added to each well to reach a final concentration of 1 μg/ml. The 96-well plate was incubated one last time for 10 min at 37°C in 5% CO_2_ incubator. Each terpene concentration was tested in triplicates on the same 96-well plate.

### Flow Cytometry Parameters

Microcapillary flow cytometry system Guava® EasyCyte^TM^ 12HT (Merck Millipore, Darmstadt, Germany) with blue laser (488 nm) was used for the acquisition of the *in vitro* inflammation assay. The detection antibody of the assay contains *R*-phycoerythrin; thus, yellow fluorescence allows to tract the concentration of TNF-α secreted by the cells (F cell %, Figure 2A). Dead cells were detected by the red fluorescence of propidium iodide (F cell %, Figure 2D). The mean flow velocity used was 35 μl/min. Each well was agitated for 10 s before each analysis using a rotatory agitator. Between each sample, the microcapillary and the agitator were washed. Data were treated using GuavaSoft (InCyte 3.1.1.) software.

### Animals

Male Wistar rats (300 g; Janvier Labs, Le Genest St. Isle, France) were used for this study. They were housed in groups of 3 or 4 under standard conditions (room temperature, 22°C; 12/12 h light/dark cycle) with *ad libitum* access to food and water and behavioral enrichment. All animals were manipulated and habituated to the tests and to the room for at least 2 weeks. All behavioral tests were done during the light period (i.e., between 7:00 and 19:00). A total number of 109 rats were used in this study (+1 who died during the procedure due to an abrupt overdose of isoflurane caused by a technical problem). They were divided into two main groups. The 91 rats that passed all three analgesic tests, calibrated forceps (mechanical sensitivity), plantar test (warm thermal sensitivity), and acetone test (cold thermal sensitivity) after one injection of monoterpene, composed a first group. The 18 rats that passed the two locomotor tests (rotarod assay and the beamwalk test) after one injection of monoterpenes composed a second group. The precise composition of the first group is as follows: CFA + Vehicle *n* = 8; CFA + Menthol (10 mg/kg) n = 6; CFA + Menthol (50 mg/kg) *n* = 6; CFA + Menthol (100 mg/kg) *n* = 8; CFA + Menthol (150 mg/kg) *n* = 7 (+1 how died); CFA + Pulegone (10 mg/kg) *n* = 6; CFA + Pulegone (50 mg/kg) *n* = 6; CFA + Pulegone (100 mg/kg) *n* = 8; CFA + Pulegone (150 mg/kg) *n* = 8; NaCl + Menthol (100 mg/kg) *n* = 8; NaCl + Menthol (150 mg/kg) *n* = 6; NaCl + Puleonge (100 mg/kg) *n* = 8; NaCl + Pulegone (150 mg/kg) *n* = 6. The precise composition of the second group is as follows: Vehicle n = 6; Menthol (150 mg/kg) *n* = 6; Pulegone (150 mg/kg) *n* = 6.

### CFA Model of Inflammatory-Induced Hypersensitivities

To induce a peripheral inflammation, 100 μl of complete Freund adjuvant (CFA; Sigma, St. Louis, MO) was injected in the right hindpaw of the rat. All CFA injections were performed under light isoflurane anesthesia (3%). Edema was quantified by measuring the width of the dorsoplantar aspect of the hind paw before and after the injection of CFA with a caliper.

### Behavioral Testing

*Mechanical hyperalgesia.* In all experimentation, to test the animal mechanical sensitivity, we used a calibrated forceps (Bioseb, Chaville, France). Briefly, the habituated rat is loosely restrained with a towel masking the eyes in order to limit stress by environmental stimulations. The tips of the forceps are placed at each side of the paw and a graduate force is applied. The pressure producing a withdrawal of the paw corresponded to the nociceptive threshold value. This manipulation was performed three times for each hindpaw, and the values were averaged.

*Thermal hot hyperalgesia.* To test the animal heat sensitivity, we used the Plantar test with Hargreaves method (Ugo Basile, Comerio, Italy) to compare the response of each hindpaw between healthy animals (unilateral intraplantar NaCl injection) and animals that received unilateral intraplantar CFA injection. The habituated rat was placed in a small box. We wait until the animal is calmed and then we exposed the hindpaw to a radiant heat. The latency time of paw withdrawal was measured. This manipulation was performed three times for each hindpaw, and the values were averaged.

*Thermal cold hyperalgesia.* To test the animal cold sensitivity, we used the acetone test to compare the response of each hindpaw between healthy animals (unilateral intraplantar NaCl injection) and animals that received unilateral intraplantar CFA injection. The habituated rat was placed in a small box, and once the animal is calm. Then, we put a drop of acetone (≥99%, Fisher Chemical) (between 50 and 100 µl) on the top of the hindpaw through a filed and curved needle without touching the hindpaw, and we scored the response of the animal during 20 s 0 no response, 1 a short response or fast movement of the hindpaw (<2 s), 2 a longer response (>2 s), and 3 licking of the hindpaw. This manipulation was performed three times for each hindpaw, and the values were summed.

*Rotarod test.* We used the Rotarod (IITC Life Science, Woodland Hills, CA). The speed of the roll increased progressively from 5 to 20 rpm in 4 min. The time spend by the animal on the rotarod before falling was measured. This test was repeated twice for each animal, and the values were averaged.

*Beam walk test.* The time needed by the rats to cross the beam (PVC bar of length: 130 cm; width: 4 cm, placed at 80 cm from the floor) was recorded. This test was repeated three times for each animal, and the values were averaged.

### Statistical Analysis

Data are expressed as mean ± standard error of the mean (SEM). Statistical tests were performed with GraphPad Prism 7.05 (GraphPad Software, San Diego, California, United States). Sigmoid dose-response curves and EC_50_ values were calculated with the following equation:

$$Y=\mathrm{Bottom}+\left(\mathrm{Top}-\mathrm{Bottom}\right)/(1+10^{\wedge }(\left(\mathrm{Log}EC_{50}-X\right)*\mathrm{HillSlope}$$ (1)

with Y the response, X the concentration, Top (resp. Bottom) the plateaus at the top (resp. bottom) of the sigmoid in the units of the *y* axis, Hillslope the steepness of the curve, and EC_50_ the concentration that gives a concentration halfway between bottom and top.

Statistical analysis was performed using Student’s *t*-test with a significance level of *α* = 0.05, meaning that differences between the negative control and the sample were considered significant when *p* < 0.05. Results were considered to be statistically significant if *p*-values were below 0.05 (*), 0.01 (**), and 0.001 (***).

For behavioral tests, data were analyzed using repeated-measures two-way ANOVA for the time course experiments, with the following factors: treatment (between) and time (within); when four groups (treatment) were compared, the Dunnett’s test was used for *post-hoc* multiple comparisons between individual groups, and when two groups (treatment) were compared, the Sidak’s test was used for *post-hoc* multiple comparisons between individual groups. To compare dose-response results, a Shapiro-Wilk normality test was performed to evaluate the hypothesis of normality, and then we performed a nonparametric Kruskal-Wallis test supplemented with a *post-hoc* Dunn’s test for multiple comparisons. For the comparison of each treatment depending on the time (0 vs. 40 min post i.p.), a Shapiro-Wilk normality test was performed. When the normality test was passed, we used the paired *t*-test, and when the normality test did not pass, we used the Wilcoxon matched-pairs signed-rank test. For the area under the curve (AUC), we tested the hypothesis of normality with a Shapiro-Wilk normality test. When it passed the normality test, we used an ordinary one-way ANOVA completed with a Holm-Sidak’s test for *post-hoc* multiple comparisons. Otherwise, we performed a nonparametric Kruskal-Wallis test supplemented with a *post-hoc* Dunn’s test for multiple comparisons. Results were considered to be statistically significant if *p*-values were below 0.05 (*), 0.01 (**), and 0.001 (***).

## Data Availability

The original contributions presented in the study are included in the article/Supplementary Material, Further inquiries can be directed to the corresponding author.
