# Supplementary material for: Anti-Hyperalgesic Properties of Menthol and Pulegone
Source: Front Pharmacol. 2021 Nov 30;12:753873. doi: 10.3389/fphar.2021.753873 (PMC8670501; doi:10.3389/fphar.2021.753873)
Supplement: Supplementary file 1 [file DataSheet1.pdf]

A

Compound	RI	Extract composition (%)	
		Calamintha nepeta (L.) savi	*Mentha piperita* (L.)
Camphene	1085	-	<0.01
β-Pinene	1119	0.35	-
3-Carene	1148	-	<0.01
Limonene	1186	-	0.57
Isomenthone	1468	8.61	1.74
Menthone	1481	0.61	9.51
Linalool	1540	-	<0.01
Menthyl acetate	1564	-	5.07
Isopulegone	1567	0.37	-
Caryophyllene	1603	1.95	0.80
Menthol	1630	4.70	42.85
Pulegone	1638	49.41	-
α-Terpineol	1674	<0.01	<0.01
Germacrene D	1690	0.94	1.58
Geraniol	1842	1.17	-

Figure S1

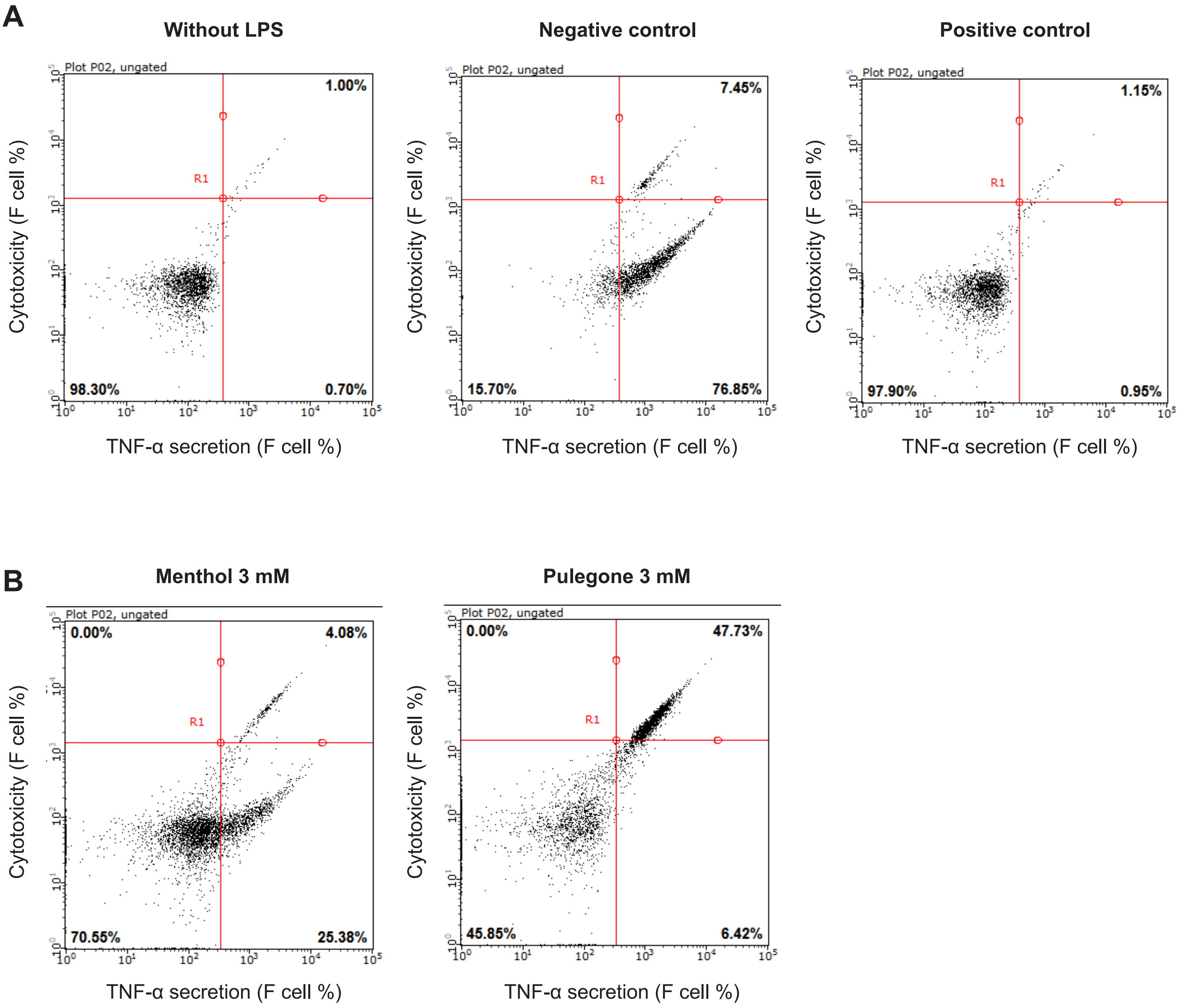
A
Without LPS
Negative control
Positive control
Plot P02, ungated
R1
1.00%
98.30%
0.70%
7.45%
15.70%
76.85%
1.15%
97.90%
0.95%
Cytotoxicity (F cell %)
TNF-α secretion (F cell %)
B
Menthol 3 mM
Pulegone 3 mM
0.00%
4.08%
70.55%
25.38%
0.00%
47.73%
45.85%
6.42%

Figure S2

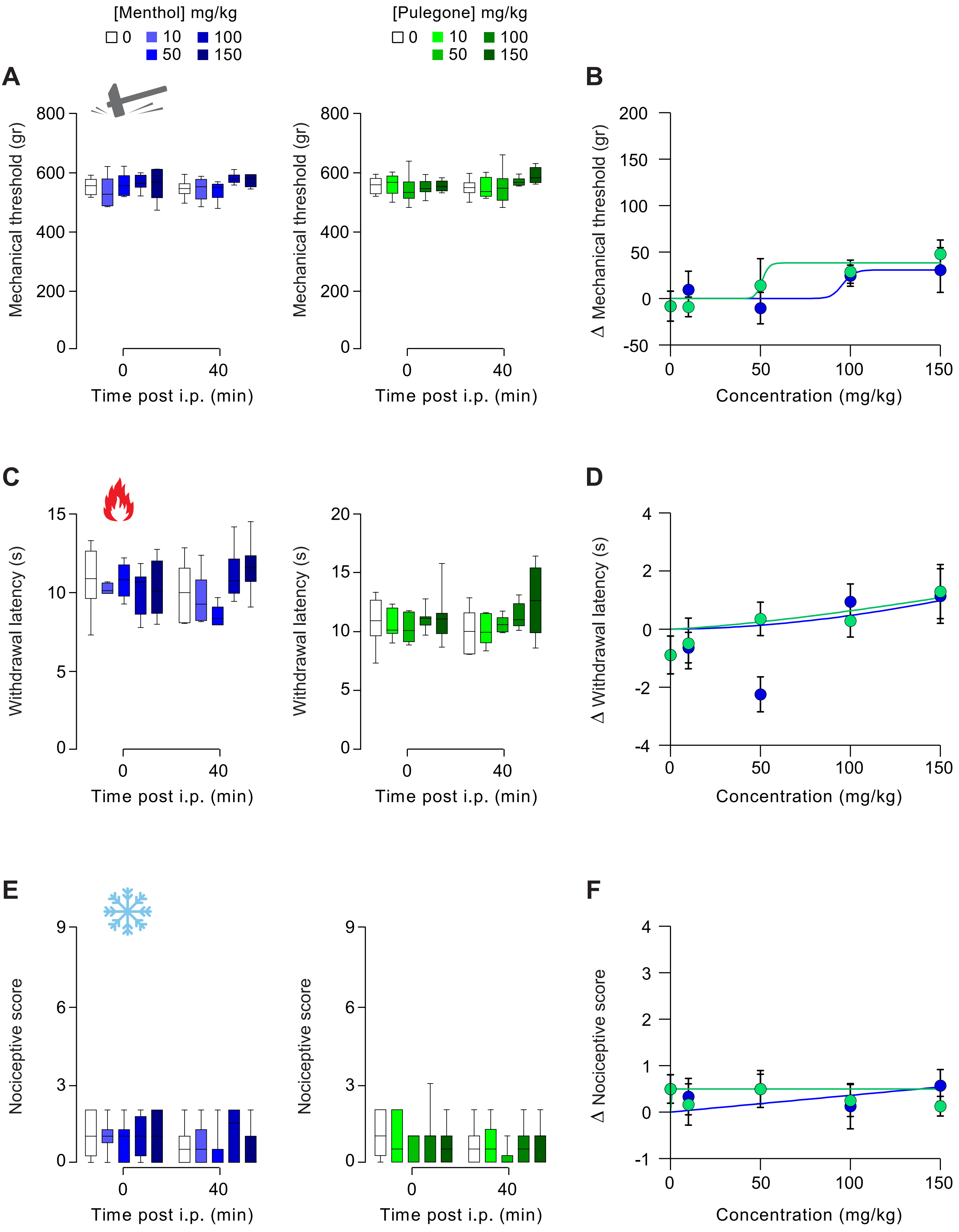
[Menthol] mg/kg
0 10 100 50 150
[Pulegone] mg/kg
0 10 100 50 150
A
B
C
D
E
F
Mechanical threshold (gr)
Time post i.p. (min)
Δ Mechanical threshold (gr)
Concentration (mg/kg)
Withdrawal latency (s)
Δ Withdrawal latency (s)
Nociceptive score
Δ Nociceptive score

Figure S3

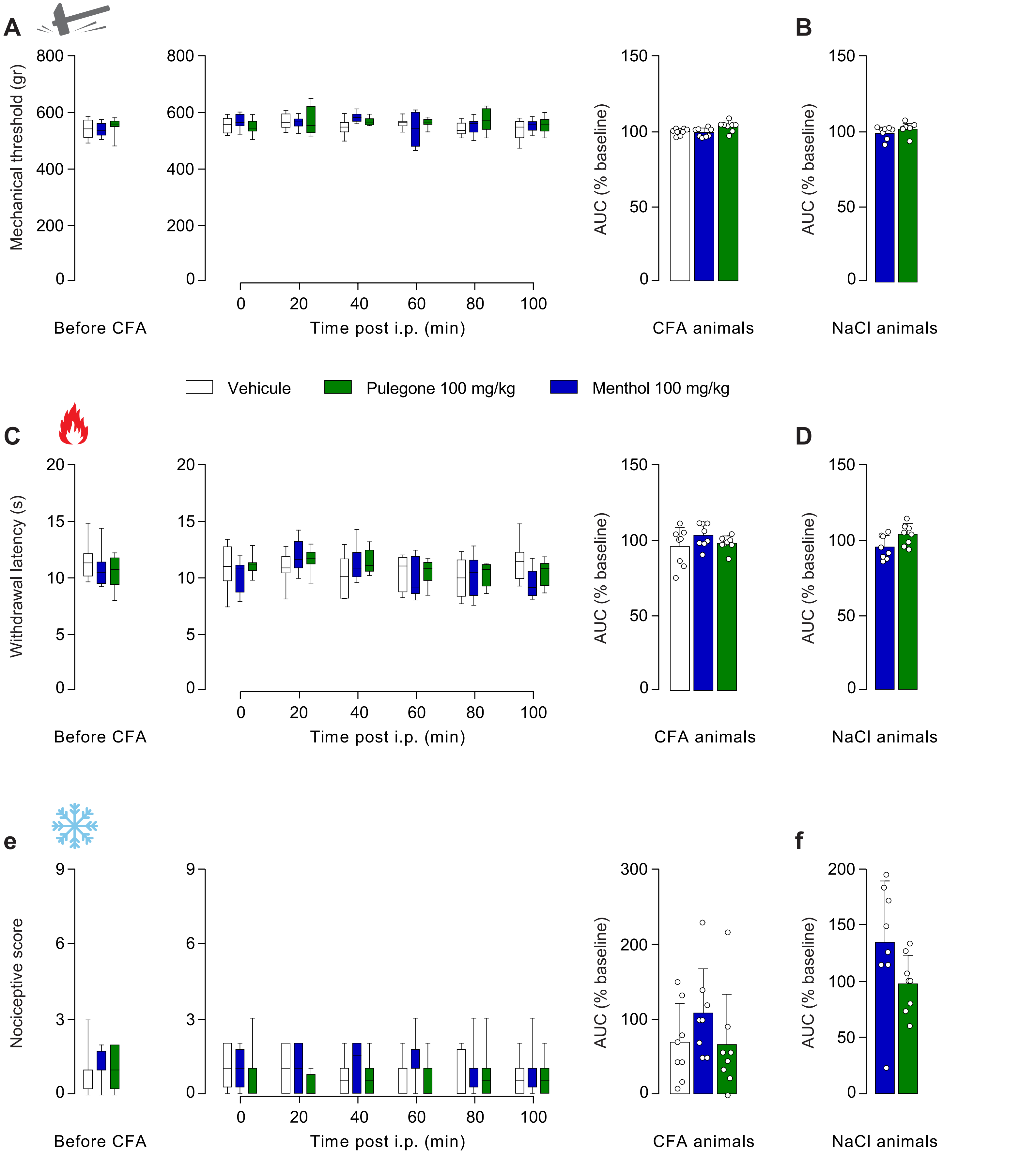
A
B
Mechanical threshold (gr)
AUC (% baseline)
Before CFA
Time post i.p. (min)
CFA animals
NaCl animals
Vehicule
Pulegone 100 mg/kg
Menthol 100 mg/kg
C
D
Withdrawal latency (s)
e
f
Nociceptive score

Figure S4

SUPPLEMENTARY FIGURES

Figure S1: Composition of the *C. nepeta* and *M. piperita* extracts obtained by PLE and analyzed by GC-MS (RI: Retention Index).

Figure S2: Cytograms of THP-1 cells for the evaluation of the inhibition of TNF-α secretion and the cytotoxicities for the controls, the standards used to determine Figure 2. The cytograms are divided in 4: lower left corresponds to inactivated cells (low yellow fluorescence, control without LPS-stimulation), lower right corresponds to activated cells (higher yellow fluorescence with the maximum for the negative control) and upper right corresponds to dead cells (high red fluorescence).

Figure S3. Dose-response of the analgesic properties of menthol and pulegone on CFA-induced inflammatory pain model – contralateral hindpaw. Effect of menthol 10 (n = 6), 50 (n = 6), 100 (n = 8) and 150 mg/kg (n = 7) and pulegone 10 (n = 6), 50 (n = 6), 100 (n = 8) and 150 mg/kg (n = 8) or its vehicle (n = 8) or their vehicle (n = 8) measured 40 min after i.p. injection on mechanical (**a**), thermal heat (**c**) and thermal cold (**e**) sensitivities. Dose-response distribution fits for menthol and pulegone on mechanical (**b**), thermal heat (**d**) and thermal cold (**f**). Data are expressed as mean ± SD. Asterisks indicate statistical significance (** $p < 0.01$; * $p < 0.05$) using a paired t test or a Wilcoxon test, depending on the data’s normal distribution.

Figure S4. Time-course of the analgesic properties of menthol and pulegone on CFA-induced inflammatory pain model – contralateral hindpaw. Baseline, time-course and relative-to-baseline AUC (%) of the effects of i.p. menthol 100 mg/kg (n = 8), pulegone 100 mg/kg (n = 8) or the vehicle (n = 8), on CFA-induced mechanical (**a**), thermal heat (**c**) and thermal cold (**e**) contralateral hindpaw sensitivities. Relative-to-baseline AUC (%) of the effects of i.p. menthol 100 mg/kg (n = 8), pulegone 100 mg/kg (n = 8) on mechanical (**b**), thermal heat (**d**) and thermal cold (**f**) sensitivities of NaCl-contralateral hindpaw. Data are expressed as mean ± SD.
